# Concomitant Expression Evolution of Cell Wall Cytoskeletal Geneic Triad(s) Controls Floral Organ Shape and Fiber Emergence in Cotton (*Gossypium*)

**DOI:** 10.3389/fpls.2022.900521

**Published:** 2022-05-20

**Authors:** Dhananjay K. Pandey, Vijay Kumar, Bhupendra Chaudhary

**Affiliations:** ^1^Amity Institute of Biotechnology, Amity University Jharkhand, Ranchi, India; ^2^Department of Botany, Shivaji College, University of Delhi, New Delhi, India; ^3^School of Biotechnology, Gautam Buddha University, Greater Noida, India

**Keywords:** cotton, gene expression, evolution, cell wall genes, floral/fiber development

## Introduction

**FIGURE 1:**
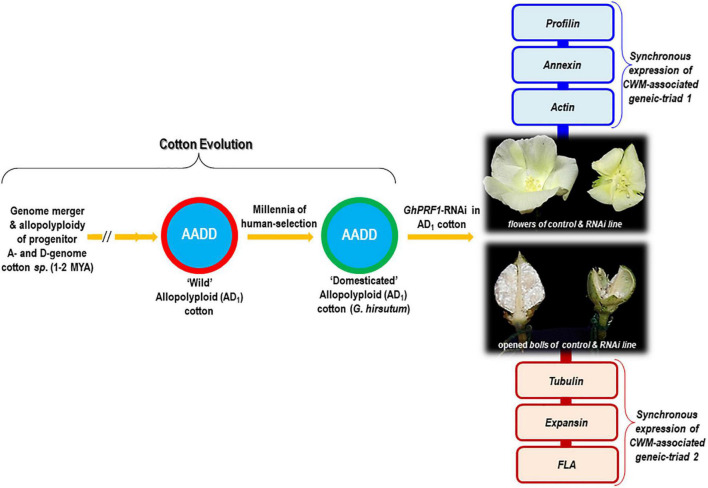
Summarized representation of cell wall-modifying (CMW) gene expression evolution accompanying the evolutionary trajectory of genomic merger and allopolyploid formation in cotton. RNAi of the *GhPRF1* gene showed abnormal floral shapes and reduced fiber development in mature bolls. Relative temporal expression of the “*GhPRF–GhAnnex–GhAct*” and “*Tub-Exp-FLA1*” geneic-triads at the 5-, 10-, and 20-dpa stages of fiber development in the Coker 310 cultivar exhibits synchronous expression patterns and highlights their fine-scale coordination during floral and fiber development.

Modern single-celled, long, and spinnable cotton fibers are the result of allopolyploidy between A- and D-genome diploid species of cotton and millennia of selective breeding under domestication (Figure 1). *Gossypium hirsutum* is one of the domesticated allopolyploid lineages and is prevalent in cotton crop fields worldwide (Wendel and Cronn, 2003). Generally, cotton fibers are elongated and thickened epidermal cells of developing ovules, which undergo four tightly regulated stages during their development, namely, initiation, elongation/extension, secondary cell wall synthesis, and maturation (Haigler et al., 2012). The comparative transcriptome sequencing of wild and domesticated species reveals that the development of fiber initiation and elongation is an extraordinarily dynamic and complex process in which more than half of the genome is expressed in any particular stage of fiber development (Yoo and Wendel, 2014). Evidently, the *morpho*-evolution of fiber cells in domesticated cotton species is characterized by large transcriptomic biases, mostly in response to hormone signaling genes, antioxidant genes, and cell wall-modifying (CWM) genes (Chaudhary et al., 2008). The latter class of genes is responsible for (de)-polymerization of actin during fiber initiation and elongation, which is mediated by transcriptionally hyperactive CWM *profilin* (*PRF*) genes (Bao et al., 2011). PRFs are extremely conserved and ancient proteins present in viruses, flagellated prokaryotes, cyanobacteria, bacteria, and animalia and plantae kingdoms (Pandey and Chaudhary, 2017). Apparently, the *GhACT1* gene responsible for constructing the actin cytoskeleton network is abundantly expressed in developing fiber cells, in particular during fiber elongation (Li et al., 2005). Mutation in the *GhACT1* gene leads to formation of disrupted F-actin filaments and disordered cytoskeleton in elongating fiber cells (Thyssen et al., 2017). Additionally, the transcriptional dynamics and functional attributes of different CWM genes have also been investigated, which includes Ca^2+^dependent phospholipid-binding *annexin* genes (*GhAnn2*, *AnxGb6*, and *GhFAnnxA*) regulating the rate of Ca^2+^ flux and signaling mechanisms, rate of fiber cell polar extension and secondary cell wall synthesis through its interaction with actin filaments (Wang et al., 2010; Zhou et al., 2011; Tang et al., 2014; Zhang et al., 2016); β*-tubulin* gene (*GhTub1*) involved in the synthesis and rearrangement of microtubules required for cytoskeletal dynamicity during fiber formation (Li et al., 2003; Chen et al., 2021); *expansin* (*GhEXPA8*) gene stimulating cell wall loosening and extension during fiber cell elongation (Bajwa et al., 2015; Lv et al., 2020); and Fasciclin-Like Arabinogalactan (*GhFLA1*) protein responsible for the activation of primary cell wall biosynthesis genes in fiber initiation and elongation processes (Huang G. Q. et al., 2013). As a result of transcriptional loss of the domestication-driven CWM-associated *profilin* structural gene (*GhPRF1*), the cotton plant experiences severe developmental abnormalities in floral organs, predominantly due to disruption of coordinated gene expression profiles of CWM-gene clusters (triads) in the cellular milieu rather than single target gene silencing (Pandey and Chaudhary, 2021). This article presents a novel perspective on the synchronized gene expression evolution of CWM genes during floral, fiber, and boll development in cotton.

## Expression Evolution of Cell Wall-Modifying-Associated “*profilin*” Genes During Fiber Development

Under cotton domestication, the selection of superior fiber traits in domesticated forms have led to the development of long and spinnable fiber phenotypes from the inferior wild short fuzz. Comparative temporal genomics of fiber development in wild and domesticated diploid and allopolyploid cotton species has identified CWM-associated cytoskeletal *profilin* (*PRF*) gene family members that are preferentially expressed (>400-fold) during emergence and extension of domesticated fiber initials (Bao et al., 2011). The functional characterization of *PRF* genes has demonstrated its direct role in the intricate process of fiber development and the regulation of floral development by activating various signaling pathways (Pandey and Chaudhary, 2016, 2021). The spatiotemporal expression analysis of *PRF* genes among vegetative, floral, and various stages of fiber development has identified *PRF* transcript abundance in developing fibers, particularly in fiber elongation stage (10 days post anthesis, dpa). Interestingly, increased *PRF* transcription exhibits proportional polymerization of F-actin levels in 10-dpa fiber tissues of different cotton cultivars, followed by 5- and 20-dpa fibers (Pandey and Chaudhary, 2019). This is attributed to the strong *PRF* genes’ expression-mediated F-actin polymerization and bundling during fiber elongation. Similarly to *PRF* genes, *annexin*, *tubulin*, and *FLA* genes are also expressed in developing fiber cells and are critical for fiber extension, secondary cell wall formation, and actin filament rearrangement (Huang Y. et al., 2013; Zhang et al., 2016). Hence, cell wall structural proteins, together with several glycoproteins and enzymes, form a rigid matrix of cellulose (Keegstra, 2010), and CWM genes contribute to maintaining a dynamic cell wall structure during fiber development.

## RNAi of *PRF* Genes Exhibits Anomalous Floral Organ Shape and Reduced Fiber Elongation

The constitutive reduction in the transcription of domestication-driven *PRF1* gene in cotton (*GhPRF1*) shows up to 40% less secondary branches and floral buds per transgenic plant compared to untransformed plants (Pandey and Chaudhary, 2021). Independent *GhPRF1*-RNAi lines exhibit floral organ abnormalities and shorter fiber lengths. Anthers are disoriented with shortened staminal tube, disorganized style protrusions, aberrant stigma tips, inadequate staminal tube growth, reduced pollen viability, and delayed and decreased fiber synthesis on the ovular surface. Most flowers fail to form seeds, and only a few produces underdeveloped bolls with stunted ovules and seeds. On the contrary, fiber-specific silencing of the *GhPRF1* gene shows normal floral organ development but reduced emergence of fiber initials on the ovule surface. Together, the constitutive- and fiber-specific RNAi of *PRF* genes in cotton modulates the expression of *actin* and *annexin* genes, which also significantly influence the *morpho*-appearance of floral organs and fibers (Pandey and Chaudhary, 2019, 2021; Figure 1). These observations suggest that abnormal floral shapes among constitutive RNAi lines are precisely the effect of reduced *PRF* transcript levels and not a consequence of transgenesis. Moreover, the transcriptional loss of *PRF* gene(s) interfered with the synchronization of gene expression ratios in the “*profilin–annexin–actin” (GhPRF1:* Accession No. EF143832; *GhAnnex3:*Accession No. JX897059; and *GhAct1:* Accession No. AY305723) and “*tubulin–expansin–FLA1”* (*GhTub1:* Accession No. AF484959; *GhExp1:* Accession No. DQ204495; and *GhFLA1:* Accession No. EF672627) geneic triads, which may account for the observed phenotypic anomalies. Future experiments in this area would provide important insights into the strategic utilization of co-expressed CWM genes for enhanced agronomically important fiber-associated traits.

## Synchronized Transcriptional Dynamics of Cell Wall-Modifying Geneic Triads Regulates Floral Organ Shape and Fiber Architecture

Previously, temporal transcriptional biases in CWM-associated genes during vegetative and fiber development were investigated among diverse cotton cultivars, and interestingly, synchronous expression dynamicity and coordinated expression patterns were prominent among CWM-associated “*profilin–annexin–actin*” and “*tubulin–expansin–FLA1*” geneic-triads (Pandey and Chaudhary, 2019). Regardless of the cultivar’s genetic background, synchronous expression profiles of the *GhPRF–GhAnnex–GhAct* triad have been observed in root tissues, hypocotyl, cotyledons, cotyledonary callus, and leaf tissues. Simultaneously, transcriptional comparisons between *GhPRF*s-associated CWM-*GhAnnex* and -*GhACT* genes in developing fibers also demonstrate a highly significant association (correlation coefficient *r* = 0.95–1) across geneic comparisons. Similarly, fold-expression variations in actin-polymerizing *GhPRF*s in different stages of fiber development are correlated with GhACT bundle formation. In addition, RNAi-mediated constitutive suppression of domestication-driven *GhPRF1* gene expression in cotton transgenics results in significant changes in CWM-*GhAnnex* and -*GhACT* gene expression profiles. At protein level, yeast two hybridization and BiFC based analyses indicate that the *AnxGb6* homodimer interacts strongly with ACT protein resulting in enhanced F-actin accumulation in fibers and regulate fiber elongation process in cotton (Huang Y. et al., 2013). The transcriptional silencing of cotton *PRF* genes reveals a strong expression correlation among these genes, which has a direct impact on cotton organ development. In response to *GhPRF1* expression reduction in transgenic cotton, *GhAnnex3* and *GhACT1* expression significantly increases. Such synchronized expression patterns are prominent in floral and fiber tissues. Notably, the *GhAnnex3* and *GhAct1* genes are significantly downregulated in deformed stamen tissues of *GhPRF1*-RNAi lines (Pandey and Chaudhary, 2019). Thus, *GhPRF–GhAnnex–GhAct* triad gene expression profiles are observed to be highly coordinated in their temporal patterns with intriguing interactions in various cellular contexts and cell types. Furthermore, the synchronized patterns of *GhPRF–GhAnnex–GhAct* triads coincide with the “tubulin *(Tub)*-expansin *(Exp)*-Fasciclin-Like Arabinogalactan-Protein (*FLA1*)” structural geneic triad during the emergence of cotton fibers. This triad mainly includes the *Tub* gene, which is responsible for enhanced and precise assembly of microtubules (Whittaker and Triplett, 1999; Mujahid et al., 2016); the *Exp1* gene is responsible for fiber cell wall relaxation (effector) (Cosgrove, 2015); the *FLA1* gene is responsible for cell wall integrity (Huang G. Q. et al., 2013). Hence, fine-scale and synchronized transcription of CWM geneic triads is essential for maintaining floral organ shapes and fiber development in cotton.

## Conclusion

Modern forms of elongated and spinnable cotton fibers originated through the genomic hybridization between A- and D-genome progenitor cotton species, followed by the formation of allopolyploids and independent domestication of two polyploidy species. For such morphological transformation of wild fuzz into modern elongated fiber cells, various genes, and transcription factors, including CWM genes, have been recruited. Both diploid and polyploid species exhibit several hundred-fold expression evolution in actin (de)-polymerizing cytoskeletal profilin genes; and the RNAi of the *PRF* genes has profound effects on the appearance of floral organs as well as fiber architecture. A remarkable feature of the temporal expression profiles of the CWM-associated “*profilin–annexin–actin*” and “*tubulin–expansin–FLA1*” geneic triads in cotton is their coordination, with an array of fascinating interplay across a variety of cellular contexts and cell types. Both floral and fiber tissues demonstrate significant expression modulation of *actin* and *annexin* genes as a result of transcriptional loss of *PRF* genes. As a result, the concurrent transcriptional dynamics of cytoskeleton-associated structural genes in modern cotton fibers are very useful in understanding the evolutionary recruitment of CWM gene clusters for shaping the floral organs and determining fiber length (Figure 1).

## Author Contributions

BC, VK, and DP conceptualized this study. All authors have contributed in the writing and further approved this manuscript.

## Conflict of Interest

The authors declare that the research was conducted in the absence of any commercial or financial relationships that could be construed as a potential conflict of interest.

## Publisher’s Note

All claims expressed in this article are solely those of the authors and do not necessarily represent those of their affiliated organizations, or those of the publisher, the editors and the reviewers. Any product that may be evaluated in this article, or claim that may be made by its manufacturer, is not guaranteed or endorsed by the publisher.
